# A Cross-Sectional Study Comparing Placental Characteristics in Pregnancy-Induced Hypertension and Sickle Cell Anaemia

**DOI:** 10.7759/cureus.70034

**Published:** 2024-09-23

**Authors:** Ujwala Bhanarkar, Pratishtha Potdar

**Affiliations:** 1 Anatomy, All India Institute of Medical Sciences, Kalyani, Kalyani, IND; 2 Anatomy, Noida International Institute of Medical Science, Noida, IND

**Keywords:** high-risk pregnancy, histopathology, placenta, placental pathology, pregnancy-induced hypertension, sickle cell anaemia

## Abstract

Background: Placental health plays a critical role in pregnancy outcomes as it serves as the interface between the mother and fetus. High-risk pregnancies, such as those complicated by pregnancy-induced hypertension (PIH) and sickle cell anaemia (SCA), are associated with significant alterations in placental morphology and histopathology, potentially leading to adverse maternal and fetal outcomes. While previous studies have explored placental changes associated with either PIH or SCA, nobody has comparatively analysed these conditions to understand their unique and overlapping effects on placental pathology.

Objective: This study aimed to compare and contrast the morphological and histopathological changes in the placenta associated with high-risk pregnancies like PIH and SCA.

Methods: A comparative analysis was conducted using data from studies at two different tertiary care centres. The study populations included 100 pregnant women diagnosed with PIH and 56 pregnant women with SCA, alongside a matched control group of 100 healthy pregnant women for the PIH group and 56 healthy pregnant women for the SCA group. Inclusion criteria were restricted to singleton pregnancies in women aged 18 to 35 years with gestational ages ranging from 28 to 40 weeks. Following delivery, placental specimens were collected, and various parameters such as weight, volume, surface area, number of cotyledons, and umbilical cord attachment were meticulously measured. Histopathological examinations were conducted to identify specific pathological features like infarcts, calcifications, syncytial knots, and fibrin deposition. Data analysis was performed using SPSS software version 25.0 (IBM Corp., Armonk, NY, USA), with a p-value of less than 0.05 considered statistically significant.

Results: The study revealed significant differences in placental parameters between the PIH and SCA groups compared to their respective controls. Placental weight, volume, and surface area were significantly reduced in both the PIH and SCA groups, with more pronounced reductions observed in PIH (p < 0.001). The umbilical cord attachment was predominantly marginal in the PIH group (75%), compared to a central attachment in the SCA group (70%), suggesting different patterns of placental development. Histopathological analysis demonstrated a higher incidence of infarcts (65% vs. 30%), calcifications (80% vs. 45%), syncytial knots (90% vs. 50%), and fibrin deposition (70% vs. 35%) in the PIH group compared to the SCA group, indicating more severe placental pathology in PIH.

Conclusion: This study offered a comprehensive comparison of placental changes in pregnancies affected by PIH and SCA. It identified both structural abnormalities in terms of placental weight, volume, and surface area and distinctive pathological features like infarctions, calcifications, syncytial knot formation, and fibrin deposits in the placenta. When compared to control groups, these findings strongly suggest that placental dysfunction is a key contributor to the adverse outcomes associated with these high-risk pregnancies.

## Introduction

Pregnancy is a physiological state that demands significant adaptations from the maternal body to support fetal growth and development. The placenta, an essential organ of pregnancy, plays a critical role in facilitating nutrient and gas exchange between the mother and fetus, as well as in the regulation of maternal-fetal immune tolerance [1]. However, in certain high-risk pregnancies, such as those complicated by pregnancy-induced hypertension (PIH) and sickle cell anaemia (SCA), the normal development and function of the placenta may be significantly altered, leading to adverse maternal and fetal outcomes [2,3].

PIH, which includes conditions such as gestational hypertension and preeclampsia, is a leading cause of maternal and perinatal morbidity and mortality worldwide. It is characterized by elevated blood pressure after 20 weeks of gestation in a previously normotensive woman, often accompanied by proteinuria and other systemic manifestations. The aetiology of PIH remains complex and multifactorial, involving abnormal placentation, endothelial dysfunction, and an exaggerated inflammatory response [4]. Abnormalities in placental development, including reduced perfusion and ischemia, are believed to contribute to the pathogenesis of PIH, leading to placental insufficiency and subsequent complications such as intrauterine growth restriction (IUGR), preterm birth, and stillbirth [2,5].

On the other hand, SCA is a genetic disorder characterized by the presence of abnormal haemoglobin S, which leads to the sickling of red blood cells under hypoxic conditions [6]. The resulting chronic haemolysis and vaso-occlusion can cause widespread organ damage, including the placenta. Pregnant women with SCA face a significantly increased risk of complications, including preterm delivery, low birth weight, and perinatal death [7-9]. The pathophysiological mechanisms underlying these outcomes are not entirely understood but are thought to involve chronic placental hypoxia, infarction, and inflammatory processes similar to those observed in PIH.

Despite the distinct etiologies of PIH and SCA, both conditions are associated with significant alterations in placental morphology and histology. These alterations can include changes in placental weight, surface area, umbilical cord attachment, and histopathological features such as infarcts, calcifications, and syncytial knots. Such changes are not merely morphological but have profound implications for the clinical outcomes of the pregnancy, as they may impair the placenta’s ability to effectively support the growing fetus.

While previous studies have independently examined the placental changes associated with PIH and SCA, nobody has undertaken a comparative analysis of these conditions [10,11]. Understanding the similarities and differences in placental pathology between these two conditions is crucial for improving the management of high-risk pregnancies and may provide insights into the underlying mechanisms of placental dysfunction.

This study aimed to fill this gap by conducting a comparative analysis of the placental morphological and histological changes in pregnancies complicated by PIH and SCA. By comparing these two conditions, the study seeks to identify specific patterns of placental pathology that may contribute to the adverse outcomes observed in them.

Furthermore, the study explored the clinical implications of these findings, to inform more effective monitoring and therapeutic strategies for affected pregnancies.

## Materials and methods

A comparative analysis was conducted at All India Institute of Medical Sciences, Kalyani, West Bengal, for a period of three months from June 2024 to August 2024 on studies at two different tertiary care centres. The study population at the first centre included pregnant women diagnosed with PIH and the other with SCA, alongside matched control groups of healthy pregnant women. A total of 100 pregnant women diagnosed with PIH were selected, with PIH defined as a systolic blood pressure of 140 mmHg or higher and/or a diastolic blood pressure of 90 mmHg or higher on two occasions at least four hours apart after 20 weeks of gestation in a previously normotensive woman [12]. Additionally, 56 pregnant women diagnosed with SCA, confirmed through haemoglobin electrophoresis, were included at the second centre. Control groups were established by selecting 100 healthy pregnant women for the PIH group and 56 healthy pregnant women for the SCA group. The controls were matched with the study participants based on age, parity, and gestational age, ensuring comparability between the groups. The matching process involved selecting control participants within ±two years of age, same parity (nulliparous or multiparous), and within ±two weeks of gestational age of the cases to minimise confounding factors.

To address any differences in protocols between the centres, specific efforts were undertaken to standardise specimen collection and analysis. Training sessions were conducted for all personnel involved in data collection and sample handling to ensure uniformity. Following delivery, placental specimens were promptly collected from all study participants following a standardised protocol. The analysis accounted for potential confounding variables that were identified during the matching process to ensure the robustness of the results.

Inclusion criteria were restricted to singleton pregnancies in women aged 18 to 35 years with gestational ages ranging from 28 to 40 weeks. Exclusion criteria included multiple pregnancies, the presence of pre-existing medical conditions other than PIH or SCA, and a history of smoking, alcohol, or drug abuse.

Following delivery, placental specimens were promptly collected from all study participants. Various placental parameters were meticulously measured, including weight, volume, surface area, shape, number of cotyledons, and the site of umbilical cord attachment (categorized as central, marginal, or velamentous). Placental weight was determined using a calibrated digital scale, while volume was estimated via the water displacement method. The surface area was calculated assuming the placenta was a circular disc, and the number of cotyledons was counted during gross examination.

Furthermore, histological processing and examinations of the placental tissue were conducted to identify specific pathological features such as infarcts, calcifications, syncytial knots, and fibrin deposition [13]. These examinations were performed under a microscope stained with haematoxylin and eosin (H&E), and the findings were documented for statistical analysis.

Data analysis was performed using SPSS software version 25.0 (IBM Corp., Armonk, NY, USA). Continuous variables were presented as mean ± standard deviation, while categorical variables were expressed as percentages. Statistical significance was determined using the Chi-square test for categorical variables and the one-way ANOVA for continuous variables, with a p-value of less than 0.05 considered statistically significant.

## Results

The study revealed significant differences in various placental parameters between the PIH and SCA groups, as well as when compared to their respective control groups.

The study revealed significant differences in placental parameters, including weight, volume, and surface area, between the control, PIH, and SCA groups (Table 1).

**Table 1:** Comparison of Placental Parameters in Control, Pregnancy-Induced Hypertension (PIH) and Sickle Cell Anaemia (SCA) Groups. *The parameters were analyzed using the one-way ANOVA test.

Parameters	Control for PIH (Mean ± SD) (n=100)	PIH (Mean ± SD) (n=100)	Control vs PIH (p-value)*	Control for SCA (Mean ± SD) (n=56)	SCA (Mean ± SD) (n=56)	Control vs SCA (p-value)*
Weight (gms)	485.8 ± 89.07	397.14 ± 76.72	<0.001	470.0 ± 80.0	446.96 ± 15.92	0.035
Volume (cc)	459 ± 98.3	385.23 ± 75.1	<0.001	450.5 ± 95.0	400.11 ± 92.2	0.041
Surface Area (cm²)	191.04 ± 33.16	156.6 ± 24.43	<0.001	188.5 ± 31.0	165.8 ± 28.54	0.029

Placental weight, volume, and surface area were significantly reduced in the PIH group than the SCA group compared to their respective controls with a p-value of <0.05, indicating impaired placental function and reduced placental efficiency.

Overall, these findings suggested that placental parameters differ significantly in both conditions (PIH and SCA) compared to their respective control groups, but the differences were more pronounced in the PIH group.

**Figure 1:**
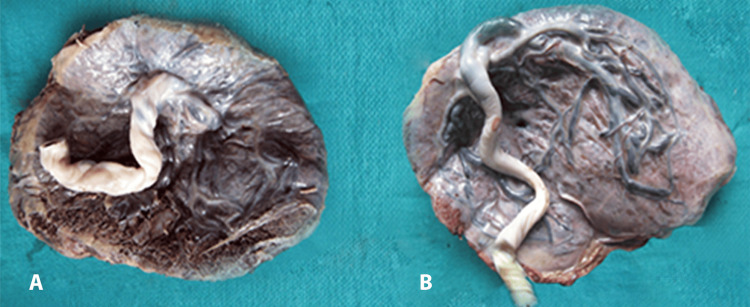
Photograph of placenta showing insertion of umbilical cord. A, Central. B, Marginal

The umbilical cord attachment was predominantly marginal in the PIH group, while it was mostly central in the sickle cell anaemia group, indicating different patterns of placental development in these conditions (Table 2, Figure 1).

**Table 2:** Comparison of Umbilical Cord Attachment PIH: pregnancy-induced hypertension, SCA: sickle cell anaemia

Group	Central (%)	Marginal (%)	Velamentous (%)
Control for PIH (n=100)	74 (74.0%)	26 (26.0%)	0 (0%)
PIH (n=100)	20 (20.0%)	75 (75.0%)	5 (5%)
Control for SCA (n=56)	41 (73.2%)	15 (26.8%)	0 (0%)
SCA (n=56)	39 (70.0%)	17 (30.0%)	0 (0%)

**Figure 2:**
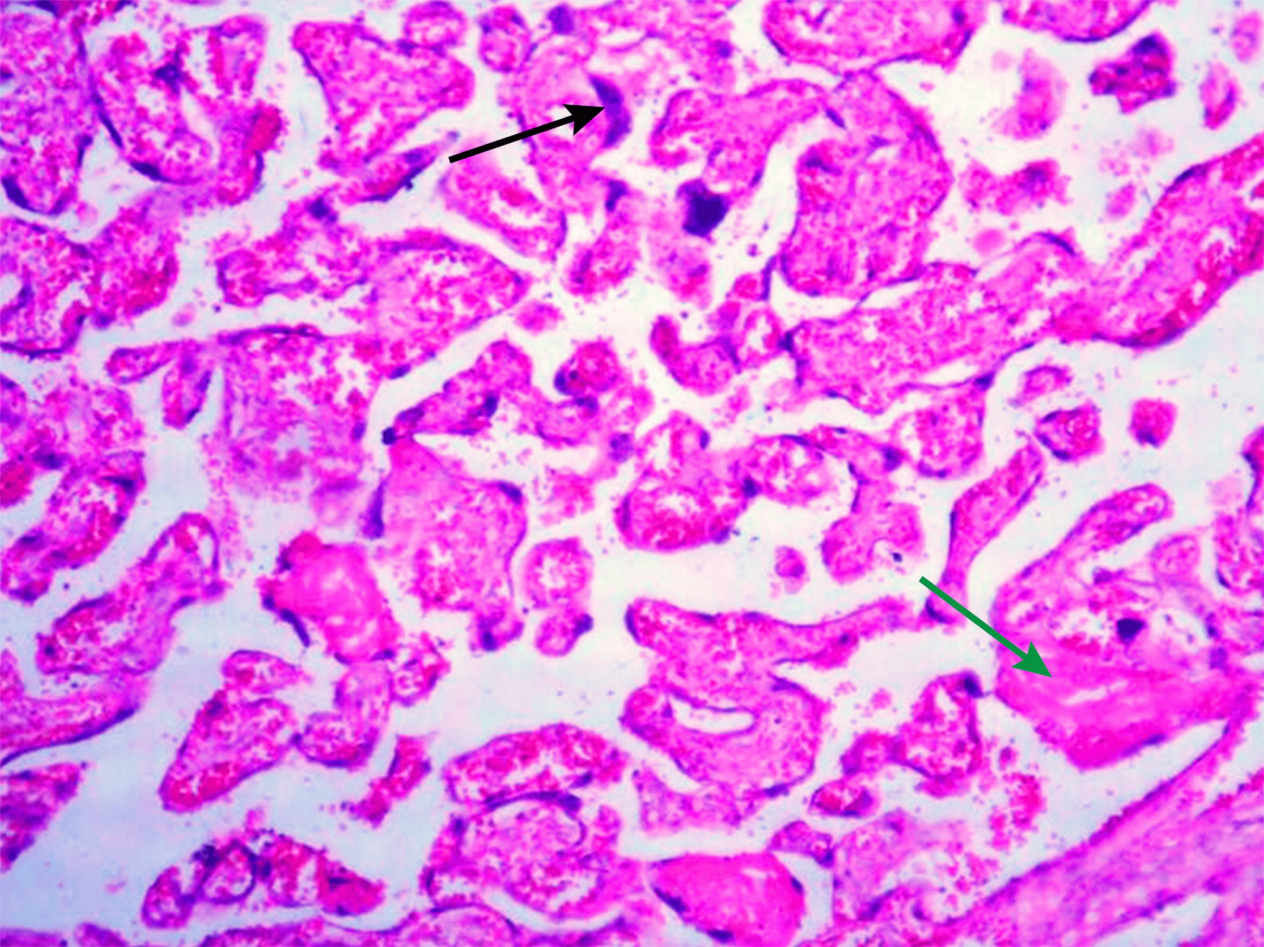
Photomicrograph of placenta showing syncytial knots (marked by black arrow) and calcification (marked by green arrow) (H&E stain 40x)

**Figure 3:**
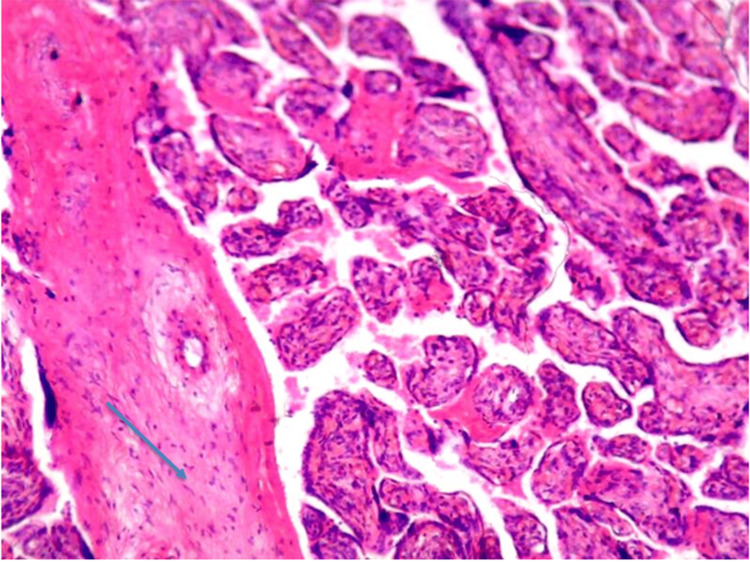
Photomicrograph of placenta showing fibrin deposition (marked by arrow) (H&E stain 40x)

The histopathological examination revealed a higher incidence of infarcts, calcifications, syncytial knots, and fibrin deposition in the PIH group compared to the SCA group, suggesting more severe placental pathology in PIH (Table 3, Figures 2, 3).

**Table 3:** Histopathological Features of Placenta *The parameter was analysed using the Chi-square test PIH: pregnancy-induced hypertension, SCA: sickle cell anaemia

Feature	Control for PIH (n=100)	PIH (n=100)	Control vs PIH (p-value)*	Control for SCA (n=56)	SCA (n=56)	Control vs SCA (p-value)*
Infarcts	13 (13%)	65 (65%)	<0.001	7 (12.5%)	17 (30%)	0.009
Calcifications	19 (19%)	80 (80%)	<0.001	11 (19.6%)	25 (45%)	0.023
Syncytial Knots	26 (26%)	90 (90%)	<0.001	14 (25%)	28 (50%)	0.015
Fibrin Deposition	16 (16%)	70 (70%)	<0.001	9 (16%)	19 (35%)	0.031

## Discussion

The current study provides a detailed comparison of placental morphological and histopathological changes in pregnancies complicated by PIH and SCA. Our findings indicate that while both conditions are associated with significant alterations in placental structure and function, the nature and extent of these changes differ markedly, reflecting the distinct pathophysiological mechanisms underlying each condition.

In pregnancies complicated by PIH, we observed a significant reduction in placental weight, surface area, and volume compared to the control group. These findings are consistent with the literature, where multiple studies have documented similar reductions in placental size in hypertensive pregnancies. For instance, a study by Mehare and Kebede (2020) highlighted that the smaller placental size in PIH is often associated with increased placental infarction and calcification, both of which were prominently observed in our study [14]. The reduced placental size in PIH is generally attributed to impaired trophoblastic invasion and abnormal remodelling of the spiral arteries, leading to insufficient uteroplacental blood flow and placental ischemia [15].

Conversely, in SCA, although there was also a reduction in placental size, the decrease was less pronounced compared to PIH. However, the incidence of placental infarctions was notably higher in the SCA group. This finding aligns with the work of Baptista et al. (2019), who documented extensive placental infarction as a hallmark of sickle cell pregnancies due to recurrent vaso-occlusive crises [16]. More recent studies, such as those by Shegekar and Pajai (2024), further support the association between sickle cell disease and significant placental pathology, including increased infarctions and fibrosis [17].

The histopathological examination in our study revealed significant differences between the two groups. In PIH, we observed an increased frequency of syncytial knots, fibrin deposition, and areas of infarction, which are indicative of placental hypoxia and stress. These findings are supported by the work of Stanek (2018), who described similar alterations in hypertensive placentas and linked them to an exaggerated inflammatory response and endothelial dysfunction [4]. Additionally, Singh et al. (2018) noted that these changes are strongly associated with adverse perinatal outcomes such as IUGR and preterm birth [5].

In SCA, the placentas showed increased villous fibrosis, calcification, and infarctions. This pattern of pathology is consistent with findings from Rathod et al. (2007), who described chronic placental hypoxia and recurrent infarctions as common features in sickle cell pregnancies [18]. The increased villous fibrosis and calcification in our study suggest a chronic process of ischemia and necrosis, likely exacerbated by the recurrent vaso-occlusive episodes characteristic of sickle cell disease. Kurrey et al. (2012) also reported similar histopathological changes, emphasizing that these alterations could impair the placenta’s ability to adequately support fetal growth, leading to high rates of fetal growth restriction and perinatal mortality [19]. 

Our study also found an increased incidence of marginal and velamentous cord insertions in the PIH group compared to controls. Velamentous insertion of the cord refers to its attachment to the membranes at some distance from the edge of the placenta [20]. These abnormal cord insertions have been previously associated with adverse pregnancy outcomes, including placental abruption and preterm delivery, as described by Chhatwal et al. (2018) [21]. The abnormal cord insertions observed in our study likely reflect the underlying abnormal placentation processes in PIH, which can disrupt normal cord attachment and function.

Interestingly, despite the different etiologies of PIH and SCA, both conditions were associated with increased syncytial knotting and fibrin deposition. This suggests that placental hypoxia may be a common feature in both conditions, albeit arising from different underlying mechanisms. In PIH, hypoxia is primarily related to defective spiral artery remodelling and placental ischemia, while in SCA, it is driven by chronic anaemia and recurrent vascular occlusions. This finding is supported by studies from Shegekar et al. (2023) and Moukalled et al. (2022) who also identified syncytial knots and fibrin deposits as markers of placental hypoxia in various high-risk pregnancies [17,22].

The distinct patterns of placental pathology observed in this study have important clinical implications. In PIH, the early detection of placental insufficiency and abnormal cord insertions could inform timely interventions to prevent adverse outcomes. This is particularly critical given the strong association between these placental changes and adverse perinatal outcomes such as IUGR and preterm birth [2,5,14]. On the other hand, in sickle cell pregnancies, the management of vaso-occlusive crises and chronic anaemia may be pivotal in mitigating placental damage and improving fetal outcomes [3,8,9,11,22]. This approach is supported by clinical guidelines that emphasize the need for close monitoring and management of maternal anaemia and hypoxia in sickle cell disease.

Limitations of the study

The study is cross-sectional, capturing data at a single point in time (at delivery). A longitudinal approach following the pregnancies throughout gestation could provide more comprehensive insights into the progression of placental changes and their impact on maternal and fetal outcomes. Although efforts were made to match control groups by age, parity, and gestational age, other potential confounding factors, such as nutritional status, socioeconomic background, or comorbidities, were not controlled for, which could affect placental development and outcomes.

While the study provides a detailed comparison of histopathological features such as infarcts, calcifications, and syncytial knots, it does not explore the molecular or genetic basis of these changes, which could offer deeper insights into the pathophysiology of placental abnormalities in PIH and SCA. Although the study highlights placental changes associated with adverse outcomes, it does not provide a detailed correlation with specific clinical outcomes (e.g., perinatal mortality and neonatal intensive care unit admissions), which could further strengthen the clinical relevance of the findings. Furthermore, the study didn't investigate the abnormal implantation and adhesions of placenta such as placenta previa and placenta accreta [20].

## Conclusions

This study provided a detailed comparison of placental changes in pregnancies complicated by PIH and SCA. The analysis highlighted both structural changes (weight, volume, surface area) and unique pathological features (infarctions, calcifications, syncytial knot formation, fibrin deposits) in the placenta. Compared to control groups, these findings strongly suggest that placental dysfunction plays a significant role in the adverse outcomes associated with these high-risk pregnancies.
